# Disseminated Aspergillosis due to *Aspergillus niger* in Immunocompetent Patient: A Case Report

**DOI:** 10.1155/2013/385190

**Published:** 2013-02-28

**Authors:** Ulku Ergene, Zeynep Akcali, Demircan Ozbalci, Nalan Nese, Sebnem Senol

**Affiliations:** ^1^Department of Hematology, Medical Faculty, Celal Bayar University, Manisa, Turkey; ^2^Department of İnternal Medicine, Medical Faculty, Celal Bayar University, Manisa, Turkey; ^3^Department of Hematology, Government Hospital of Mersin, Mersin, Turkey; ^4^Department of Pathology, Medical Faculty, Celal Bayar University, Manisa, Turkey; ^5^Department of Infectious Diseases and Clinical Microbiology, Medical Faculty, Celal Bayar University, Manisa, Turkey

## Abstract

Invasive aspergillosis is a major cause of morbidity and mortality in immunocompromised patients. Many cases of pulmonary, cutaneous, cerebral, and paranasal sinus aspergillosis in immunocompetent patient were defined in literature but disseminated aspergillosis is very rare. Here we present an immunocompetent case with extrapulmonary disseminated aspergillosis due to *Aspergillus niger*, totally recovered after effective antifungal treatment with voriconazole.

## 1. Introduction

Although hundreds of *Aspergillus* conidia survive within the inhaled air, no disease development occurs at immune competent individuals [1]. Immunosuppression increases the risk of dissemination of the *Aspergillus* to all of the solid organs via circulation or by direct tissue invasion [2]. In the experimental models of aspergillosis by hematogenous infection, the microorganisms tend to invade the liver and the spleen especially [3]. However, extra-pulmonary aspergillosis occurs in 25–60% of cases and may involve the central nervous system, liver, skin, and gastrointestinal system [4–6]. In the immunocompetent cases, disseminated aspergillosis is infrequently reported [7]. Herein we would like to present an immunocompetent case with extra-pulmonary disseminated aspergillosis totally recovered after effective antifungal treatment with voriconazole. 

## 2. Case

**Figure 1:**
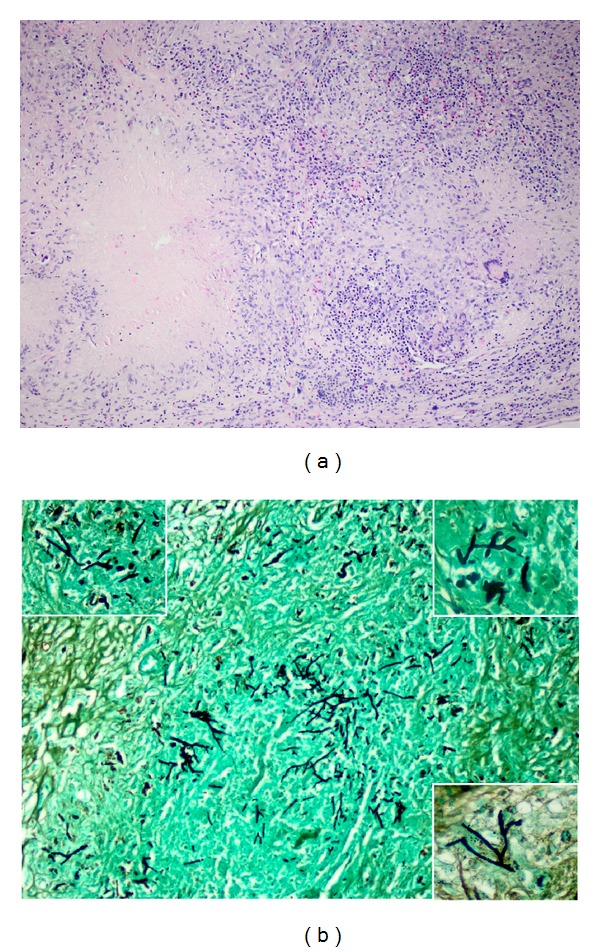
Excisional biopsy material of the cervical lymph node: (a) hematoxylin and eosin and (b), Gomori methenamine silver stain; caseation necrosis and giant cell granulomas with several fungus hyphae, they were morphologically branching hyphae with right/acute angle.

52-year-old woman was referred to our emergency service with severe abdominal pain. She also complained of progressive jaundice and weight loss for three months. She had no history of immunosuppressive disease or treatment. Physical examination revealed jaundice of the sclera and the entire skin, hepatomegaly, massive and tender splenomegaly, and enlargement of the lymph nodes at occipital, axillary, and the inguinal sites and normal body temperature. Laboratory examination showed that erythrocyte sedimentation rate (ESR) was 120 mm/h, absolute white blood count (WBC) was 15300/mm^3^, hemoglobin (Hgb) was 10.4 g/dL, and platelet (PLT) count was 443000/*μ*L. Blood smear was performed, and significant eosinophilia (32%) was observed with degranulation and nuclear irregularities. Alanin aminotransferase (ALT) levels were increased to 690 U/L, and the total serum bilirubin was 9.45 mg/dL. Thus abdominal ultrasound imaging was performed confirming the enlargement of the liver and the spleen (186 mm and 200 mm, resp.) and dilatation of both the intra- and extrahepatic biliary tract. As eosinophilia was observed at the peripheral blood smear, bone marrow sampling was performed. Smear of the bone marrow revealed marked eosinophilia (30%) and a normal blast ratio. No parasitic organism was observed. The bone marrow specimen was also negative for *t*(9; 22) and PGFR*α*. Total body computed tomography scans showed multiple lymphadenopathies at neck and the periportal area ascites besides hepatosplenomegaly, and there was no pulmonary involvement. Finally, trucut biopsies of the liver and excisional biopsy of the cervical lymph node were obtained. Both of them showed disseminated caseation necrosis and giant cell granulomas with several fungus hyphae and spores (Figure 1). As they were morphologically branching hyphae with right/acute angle, *Aspergillus* was considered. Consequently, voriconazole was started with 6 mg/kg twice-daily loading dose and then 4 mg/kg maintenance dose. Bone marrow and lymph node material cultures led to the identification of the agent as *Aspergillus niger*. After two-month follow-up, physical examination was normal and the laboratory values significantly decreased. As all clinical and laboratory finding were returned to normal, treatment was ceased on day 112. 

After seven months of follow-up, she had no complaints and normal total blood counts with eosinophilia being 3.4%. Eighteen months later, she is still disease-free. 

## 3. Discussion

As the number of immonocompromised patients increased, the *Aspergillus* conidia became the most prevalent airborne pathogens. The members of this species are one of the major pathogens that are responsible for fatal infections at hematology and transplantation units [7]. However, invasive aspergillosis (IA) is infrequent between immunocompetent individuals. Steinbach et al. reported a multicentric cohort of 960 IA cases. In this cohort, only 17 (3.7%) cases had no risk factors for immunosuppression like malignancy or transplantation, on the other hand, all these cases were either newborn intensive care unit patients or undergone major surgery other than transplantation [8]. Our patient neither had a history of surgery nor had immunosuppressive disease or condition. Severe hepatic disease is also another risk for IA [9]. The case we present here had severe elevations of aminotransferases; however they responded to voriconazole regimen dramatically suggesting that this impairment of liver function was an outcome of IA rather than to be a predisposing factor of it. 

Involvement of the liver and spleen is seen in about 15% of cases of IA; however this is usually a result of dissemination from the lung. As the gastrointestinal tract is a well-known portal of entry for many pathogens, *Aspergillus* species is one of them [10]. Our case had lesion at thorax CT scans, but of course, *A. niger* fungemia might be originating from either the respiratory tract or gastrointestinal tract.

*A. niger* is showed to have the lowest rate of invasive disease (2.4%) between all the *Aspergillus spp. in vitro* [11]. However, clinical studies revealed lower rates showing that *A. niger* is responsible for 4–8.7% of IA cases [8]. Furthermore, survival rates seem better in patients who developed the disease with *A. niger* compared to the other *Aspergillus spp.* [7]. Our patient also had a favorable response to treatment and was infected with *A. niger*. Thus, our therapeutic success might be resulting from the pathogenic properties or the patient's compatible immune response of course. 

In an experimental study of mice, *A. fumigatus*, when given intravenously, may cause infection in both of the immunocompetent and immunocompromised ones. However, immunosuppressive mice are reported to have more frequent and more disseminated disease. Hepatic and splenic involvements were seen in all immunocompromised mice in the first 24 hours. Nevertheless, in the immunocompetent mice disease was far more limited [3]. This observation suggests that primary host defense is the major determinative factor for the severity of *Aspergillus* infection.

Diagnosis of IA relies on combination of several nonspecific clinical findings and laboratory findings. Especially for the IA of solid organs other than the lungs, careful histological examination and cultures of the tissue specimen are essential for the diagnosis [8]. In our case, observation of granulomatous inflammation and *Aspergillus* hyphae at lymph node biopsy specimen in other to rule out malign lymphoma helped us to diagnose early.

The North American Infectious Disease Society's (IDSA) Guidelines of 2008 recommend amphotericin B-deoxycholate for regions with restricted resources only, which could be the case in underdeveloped countries [12]. Liposomal amphotericin B in the daily standard dose of 3 mg/kg offers a rate of response similar to the one with voriconazole in the first-line treatment of invasive aspergillosis. Good oral and parental bioavailability voriconazole penetrates well to tissues and SSS. However, in the literature, there is no specific suggestion about disseminated aspergillosis. In our case, we preferred to use voriconazole as it has lower risk for side effects, peroral administration advantage, and successful outcomes in other IA forms. There is no consensus on the adequate duration of the treatment. Considering that hepatic involvement is generally observed with disseminated disease and the difficulty of clearance of *Aspergillus* from biliary tract [10, 12] we had continued to use voriconazole until after one month of the clinical and laboratory response. 

Increasing body of immunocompetent IA cases suggests that, when infected with sufficient amount of pathogens, all *Aspergillus* species especially *A. fumigatus *may result in disease even in the normal individuals by means of the immune system [13]. Further considerations should take place especially in the cases with fever of unknown origin.
